# Use of glycerol triheptanoate as a marker for processed animal by-products - results from 2010–2024

**DOI:** 10.2478/jvetres-2025-0069

**Published:** 2025-12-12

**Authors:** Aleksandra Grelik, Ewelina Kowalczyk, Krzysztof Kwiatek

**Affiliations:** Department of Chemical Research of Food and Feed, Department of Microbiological Research of Food and Feed, National Veterinary Research Institute, 24-100 Puławy, Poland; Department of Microbiological Research of Food and Feed, National Veterinary Research Institute, 24-100 Puławy, Poland

**Keywords:** animal by-products, flame ionisation, food and feed safety, glycerol triheptanoate (GTH), mass spectrometry

## Abstract

**Introduction:**

Whole dead animals, parts of dead animals, products of animal origin or other products derived from animals that are not intended for human consumption are animal by-products, and legislation imposes restrictions on the use of those which may pose a risk to the food and feed chain. High-risk products should only be used outside the feed chain. Unsafe by-products are distinguished from safe ones, parcel by parcel, and those with the highest harm potential are permanently marked during processing with glycerol triheptanoate (GTH) to prevent their entry into the feed chain. The legislated minimum content of this marker must be 250 mg/kg of fat. This research is on the development and validation of methods using gas chromatography with flame ionisation or with mass spectrometry for GTH detection, and also comprises a report of compliance with the GTH content threshold among samples of animal by-products.

**Material and Methods:**

Between 2010 and 2024, 2,303 samples of meat and bone meal, rendering fat, processed animal protein, soil improvers, antioxidants, feed materials and mixtures, dog chews, feathers, bird balls and unknown material of animal origin were tested. Gas chromatography was used with either flame ionisation detection or mass spectrometry.

**Results:**

Samples that did not meet the requirements under applicable law accounted for approximately 10.5% (240 samples). The highest percentage of non-compliant samples was recorded in the processed animal proteins group (20.7%). Incorrectly marked meat and bone meal and rendered fat accounted for 8% and 12% of their groups, respectively.

**Conclusion:**

Nearly 90% of the samples tested were correctly marked with GTH as required or free of it, which may indicate progress in developing effective marking technology at processing plants.

## Introduction

As more animals are slaughtered, the rendering industry grows to respond to need. The rendering industry is jointly accountable under current food law for the safety of the entire food chain. This safety encompasses animal health, the control of infectious diseases, the source of food raw materials, their processing and the final product – food (6, 7, 8, 9). Legal regulations define animal by-products (ABPs) as whole dead animals, their parts, and products of animal origin or other products derived from animals that are not intended for human consumption. The legislative framework exists because some ABPs constitute a potential source of risk if included in the food and feed chain (10). If any ABP is subjected to pressure, temperature, transformation or processing, it automatically becomes a derived product. Annex IV of Regulation (EU) No. 142/2011 sets out the rules for the processing and transformation of ABPs, codifying what derived products have undergone (5). These products are subject to exactly the same regulations as ABPs in order to ensure their safe use. Only processed animal protein the use of which poses minimal risk to the food chain is permitted for use in farm animal feed. This requirement is met by those products derived from materials which are classified as category 3 (the lowest risk), which were collected or processed under the required pressure sterilisation conditions to prevent risks to human and animal health, and which originated from approved or registered establishments appropriate to the product category concerned (10, 12). Category-3 ABPs are not intended for human consumption for commercial reasons and derive from healthy animals. Products derived from the higher-risk category-1 and -2 materials must be excluded as ingredients in farm animal feed. Therefore, it is crucial to implement methods that allow the effective withdrawal of processed animal protein products derived from category-1 and -2 ABPs that are not permitted in animal feed.

The main sources of animal by-products are the slaughter of farm animals and the processing of animal-based food raw materials. It is estimated that meat-processing plants and slaughterhouses across the European Union produce in excess of 20 million tonnes of ABP annually. In Poland, the amount of ABP currently generated is estimated at approximately 2 million tonnes per year (11). Ensuring sanitary and epizootic safety in the country has in recent years needed mass culls for combatting outbreaks of infectious diseases and has necessitated disposing of carcasses. Since the continent has been contending with the need to liquidate herds of swine and free-living wild boar due to African swine fever, slaughtering and disposing of animals has generated approximately 150,000 carcasses of suids in recent years in Poland. However, the number of birds disposed of in connection with avian influenza outbreaks in Poland is significantly higher. In 2019–2020, this number stood at 3.3 million, corresponding to approximately 10,000 tonnes, while in only the half-year January–June 2021, over 17.5 million poultry (almost 40,000 tonnes) were disposed of (11). Other significant sources of ABP include manure; expired, unsaleable or damaged food; catering waste; animal skins and wool; fish parts; dead pets and zoo animals; blood; eggs; and feathers and bristles (10).

To prevent processed products derived from category-1 and -2 materials from entering into the food and feed chain, a European requirement was introduced on 1 July 2008 to permanently mark such derived products using glycerol triheptanoate (GTH) (4). This artificial substance is a triglyceride containing three molecules of n-heptanoic acid. It is obtained from a purified fraction of crude oil boiling at 40–70°C, is commercially available and has already been used in the food industry. Glycerol triheptanoate dissolves perfectly in the fat fraction and can be used to mark fat or meal containing that fraction. Studies prior to its marker use have shown that it is not stable in raw animal by-products because the enzymes present in them degrade it. Therefore, in order to mark ABPs permanently, it is added to derived products that have undergone heat treatment at a temperature of at least 80°C, at which the enzymes are inactivated. Experiments conducted under laboratory conditions have shown that GTH can withstand steam heat treatment at 133°C, which is required for the processing of meat and bone meal derived from mammals. Detailed rules for the use of GTH as a marker are specified in Commission Regulation (EU) No. 142/2011 of 25 February 2011: ABPs derived from category-1 or -2 materials must be permanently marked with GTH after undergoing a heat treatment process which reaches a temperature of at least 80°C. The concentration of GTH required in the final product should be at least 250 mg/kg of fat and be uniform throughout its mass (5).

The first aim of this publication is to present the results of 15 years of surveillance of processed ABPs to find the GTH marker content in those required to have it and to detect GTH in products required to be free of it. The second aim is to demonstrate the effectiveness of gas chromatography (GC) methods either with flame ionisation detection (FID) or mass spectrometry (MS). The study encompassed both methods’ optimisation and validation, as well as the results of real sample analyses.

## Material and Methods

### Reagents and materials

During the methods’ optimisation and sample analyses, reagents of analytical-grade purity and chromatographic purity for GC were used. Isooctane was purchased from Merck (Darmstadt, Germany). Petroleum ether with a boiling point of 40–60°C, n-hexane, and diethyl ether, all analytical grade, were obtained from Avantor (formerly POCH, Gliwice, Poland). Reference standards of glycerol triheptanoate and 5α-cholestane were purchased from Sigma-Aldrich (Burlington, MA, USA). Additionally, the entire testing process also required the use of 3 mL solid-phase extraction (SPE) columns with 60 mg of NH2-modified sorbent purchased from Agilent Technologies (Santa Clara, CA, USA), boiling chips (Merck, Darmstadt, Germany), round-bottomed cellulose tubes in a 33 × 80 mm size (Prat Dumas, Couze-et-St-Front, France) and glass or cotton wool.

### Standard solutions

A stock standard GTH solution for GC-FID and GC-MS analysis was prepared by weighing 25 mg of the reference substance into a 25 mL volumetric flask, dissolving it in isooctane and making the solution up to the required volume with isooctane. The stock solution of the 5α-cholestane internal standard for the GC-FID analysis (0.6 mg/mL) was prepared by weighing 15 mg of the standard substance also into a 25 mL volumetric flask, dissolving it in isooctane and making the solution up to the volume with isooctane. For GC-MS, the 5α-cholestane stock solution (1 mg/mL) was prepared in the same manner as the GTH stock standard solution. Stock standard solutions were stored at 2°C–8°C for no longer than six months. The concentration of the GTH working solutions for both types of detection was 0.01 mg/mL. The working 5α-cholestane solution concentration was 0.03 mg/mL for the MS detection, and was undiluted stock solution (0.6 mg/mL) for the FID. Working solutions were freshly prepared prior to each analysis.

### Samples

In 2010–2024 a total of 2,303 samples were tested, including meat and bone meal (MBM – 1,163 samples), rendered fat (1,017 samples), processed animal proteins (58 samples), soil improvers (30 samples) and others (35 samples). Materials collectively grouped as “other” included unknown animal material (9 samples), antioxidants (3 samples), feed materials and mixtures (5 samples), dog chews (2 samples), feathers (1 sample), bird balls (1 sample), and quality control samples, which were GTH markers (14 samples) and were considered less frequently tested materials. The numbers and types of analysed samples are given in Table 1. Samples were collected as part of the Official Feed Control Plan from processing plants, warehouses and collection and transport points (505 samples); through official surveillance conducted by the Veterinary Inspectorate as part of explanatory proceedings (487 samples); in the course of carrying out the integrated Multiannual Programme for monitoring feed and food safety, animal welfare and plant and animal health (778 samples); and as commercial tests provided by processing plants and distributors as part of internal control (533 samples). The tested material was stored in closed containers at room temperature or, if it was fat, at 6 ± 2°C.

**Table 1.** Type and number of samples tested for glycerol triheptanoate marker content in Poland 2010–2024

Type of material	Meat and bone meal	Fat	Processed animal proteins	Soil improvers	Others						
					GTH marker	Unknown material of animal origin	Antioxidants	Feed materials and mixtures	Dog chews	Feathers	Bird balls
Numer of samples	1,163	1,017	58	30	14	9	3	5	2	1	1
					Total: 35						
Total	2,303										

### Sample preparation and fat extraction

Sample preparation for routine testing of MBM and PAP involved grinding the material and passing it through a 1-mm sieve. The next step was fat extraction. Samples weighing 10 g ± 0.001 g were extracted with 150 mL of petroleum ether using an Avanti automatic Soxhlet extraction apparatus (Foss Analytical, Hillerød, Denmark) continuously for 6 h, with the operating temperature maintained between 70 and 80°C. The extracted fat was then dried to constant weight. From the obtained fat, 0.5 ± 0.001 g was weighed into 5 mL volumetric flasks and dissolved in n-hexane, and the solution was made up to the volume with n-hexane.

For purification of fat solutions, SPE was performed using cartridges containing Bond Elut amino-modified sorbent (NH2) (Agilent Technologies, Santa Clara, CA, USA) preconditioned with n-hexane. A 0.2 mL aliquot of fat solution was loaded into each cartridge. Glycerol triheptanoate was eluted using an n-hexane:diethyl ether mixture (8.5:1.5, v/v). The eluates were evaporated to dryness under a nitrogen stream, and the residues were dissolved in 5 mL of GC-grade isooctane (1, 2). For rendered fat samples, which do not require a fat extraction step, the same protocol was followed as for MBM and PAP, starting from the step of weighing 0.5 ± 0.001 g into 5 mL volumetric flasks.

### Gas chromatography optimisation and analysis

Available sources indicated gas chromatography with flame ionisation or mass spectrometry detection as suitable for determining the GTH content in processed animal by-products. Therefore, these approaches were used to develop the methods used in the National Veterinary Research Institute (1, 2). The operating conditions of the apparatus were selected to achieve optimal results. Variations were made in selection of the chromatographic column and its thermostat operating programme, carrier gas flow, dosing technique and operating temperature of the injector, selection of the temperature and gas ratio for the flame ionisation detector, and equivalently in the operating parameters of the mass spectrometer. The gas chromatography system consisted of a 6890N gas chromatograph equipped with an autosampler, a split-flow gas injector and a flame ionisation detector for FID, and a 7890A gas chromatograph equipped with an autosampler, a splitflow gas injector and a 5975C single-quadrupole mass spectrometer (all from Agilent Technologies) for MS. Both systems were equipped with ChemStation data collection and processing software (Agilent Technologies). The analyses were performed using non-polar DB-5 and DB-5 MS (mass-spectrometry-compatible) chromatography columns of 30 m length with 0.25 μm film thickness and 0.25 mm internal diameter, rated for operation up to 350°C (Agilent Technologies). Initially, the analyses were performed using the GC-FID technique, then using this and GC-MS in parallel, and from 2014 only using GC-MS.

### Validation studies

To assess the applicability of the optimised methods to specific tasks, validation was carried out. The developed methods were validated in accordance with the selected requirements of European Commission Decision 2002/657/EC. The validation process involved determining the following parameters: signal linearity, working range, selectivity, repeatability, within-laboratory reproducibility and recovery. Additionally, the limit of detection (LOD) and limit of quantification (LOQ) were determined. Uncertainty was also determined during the method validation step.

Evaluation of the method’s linearity was based on the analysis of calibration curves and the assignment of an R2 determination coefficient. The calibration range of GC-FID method covered 75–600 mg/kg of fat concentrations, and the calibration curve points were 75, 150, 300, 450 and 600 mg/kg of fat. In the GC-MS method, the linearity range was slightly wider and covered 75–625 mg/kg of fat concentrations, and the calibration curve points were 75, 250, 375, 500 and 625 mg/kg of fat. Linearity was achieved if the determination coefficient was higher than 0.98.

Recovery values were calculated by comparing the concentration obtained from the MBM, PAP or rendered fat samples with the added amounts. Specific volumes of standard solutions were added to the starting material to achieve the desired concentrations relative to fat mass (75, 250, and 600 mg/kg fat for GC-FID, and 75, 250, and 625 mg/kg fat for GC-MS). Repeatability was assessed by comparing the results of six replicates prepared on the same day at three different concentrations. Within-laboratory reproducibility was assessed by spiking two other sets of blank samples at the same concentrations as for repeatability and having them analysed on different days with the same instrument but by different technicians. Standard deviations and coefficients of variation were calculated for each level. Category-3 MBM and PAP were used to determine the selectivity of the method, as well as to establish the LOD and LOQ using the signal-to-noise approach. Additionally, accuracy and precision were evaluated at the established LOQ. Measurement uncertainty was assessed using the uncertainty budget approach, which involved identifying and quantifying all sources of uncertainty that could influence the measurement result. Validation parameters were determined for MBM and rendered fat.

## Results

### Method optimisation results

The optimal operating parameters for the gas chromatograph with a flame ionisation detector and with a mass spectrometer are given in Tables 2 and 3.

**Table 2.** Optimal operating conditions for gas chromatograph and flame ionization detector

Carrier gas	Helium, flow rate 2.3 mL/min
Capillary column	DB-5, 30 m, Internal diameter 0.25 mm, film thickness 0.25 μm
Column thermostat	Initial temperature 80°C for 1 min Ramp 70°C/min to 250°C (hold for 1 min) Ramp 73°C/min to 330°C Final temperature 330°C for 7 min
Injector temperaturę	280°C
Sample injection	Split 10:1, injection volume 2 μL
Detector temperaturę	360°C
Detector gases	Hydrogen:synthetic air 1:10; 40 mL/min: 400 mL/min

**Table 3.** Optimal operating conditions for gas chromatograph and mass spectrometer

Carrier gas	Helium, flow rate 1.4 mL/min
Capillary column	DB-5 MS, 30 m, Internal diameter 0.25 mm, film thickness 0.25 μm
Column thermostat	Initial temperature 70°C for 1 min Ramp 45°C/min Final temperature 325°C for 8 min
Injector temperature	250°C
Sample injection	Split 20:1, injection volume 2 μL
Ionisation type	Electron ionisation (EI)
Dwell time	50 msec
Ion source temperaturę	230°C
Operation mode	Selected ion monitoring (SIM)
Recorded ions (m/z)	Glicerol triheptanoate - 299.1; 285.0; 228.2 Internal standard – 217.0; 372.4, 218.0
Tuning	Automatic optimisation - autotune

The combination of optimal chromatographic separation conditions, fat extraction, and sample purification resulted in methods that enabled the determination of GTH content in derived products. Example chromatograms of samples analysed using the optimised methods are shown in Figs 1a, 1b, 1c and 2a, 2b and 2c.

**Fig. 1a.**
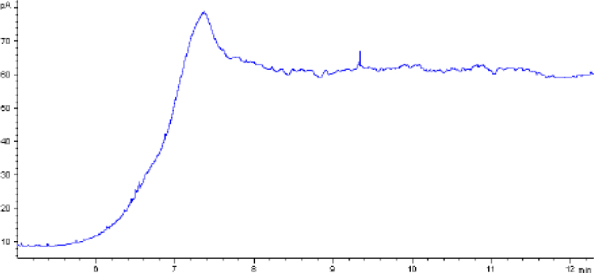
Chromatogram of a blank animal by-product sample obtained using gas chromatography–flame ionisation detection

**Fig. 1b.**
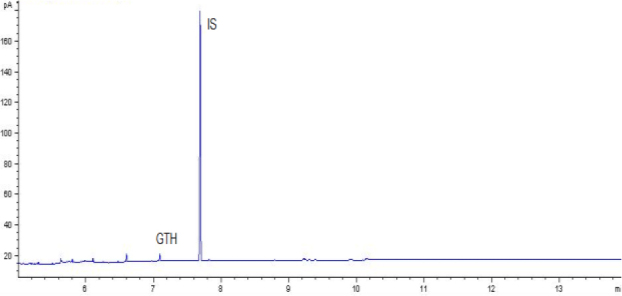
Chromatogram of a control sample with glycerol triheptanoate (GTH) content of 250 mg/kg of fat using gas chromatography–flame ionisation detection. IS – internal standard

**Fig. 1c.**
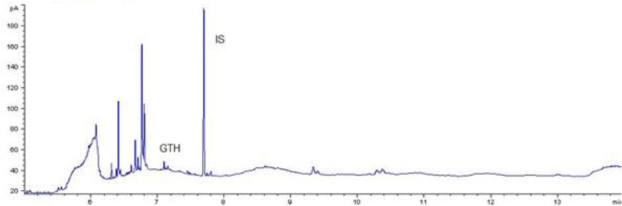
Chromatogram of a routine sample with glycerol triheptanoate (GTH) content of 440 mg/kg of fat using gas chromatography–flame ionisation detection. IS – internal standard

**Fig. 2a.**
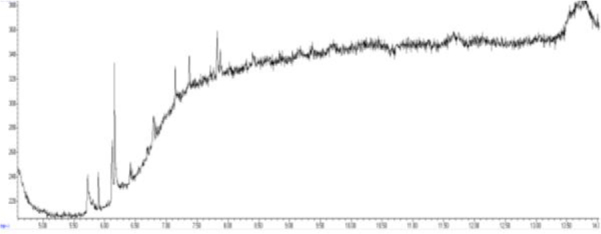
Chromatogram of a blank sample obtained using gas chromatography–mass spectrometry

**Fig. 2b.**
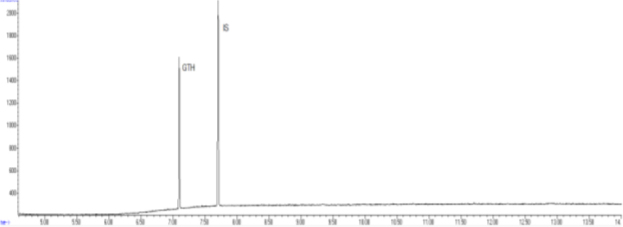
Chromatogram of a control sample with glycerol triheptanoate (GTH) content of 250 mg/kg of fat using gas chromatography–mass spectrometry. IS – internal standard

**Fig. 2c.**
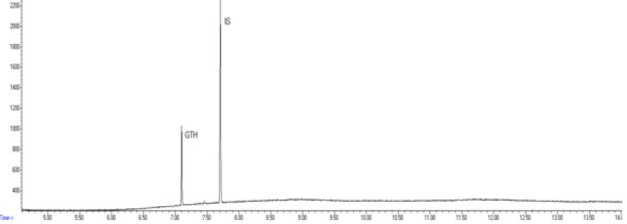
Chromatogram of a routine sample with glycerol triheptanoate (GTH) content of 160 mg/kg of fat using the gas chromatography–mass spectrometry. IS – internal standard

### Methods validation results

The method was validated in terms of linearity, working range, selectivity, repeatability, reproducibility, recovery, LOD and LOQ. Additionally, the uncertainty of the measurement was assessed. The methods’ validation results are presented in Tables 4 and 5. Linearity was confirmed from calibration curves over 75–600 mg/kg of fat for GC-FID and 75–625 mg/kg of fat for GC-MS, with coefficients of determination (R^2^) exceeding 0.99 in both cases. The method demonstrated selectivity, as no interfering peaks were observed at the retention times of GTH or the IS. For GC-FID analysis, the LODs were 28.93 mg/kg of fat for MBM and 25.07 mg/kg of fat for rendered fat, and the LOQs were 60.43 mg/kg of fat and 37.26 mg/kg of fat, respectively. The coefficients of variation for repeatability ranged from 2.64 to 8.79% and for reproducibility from 2.87 to 11.79% for MBM, and these ranged from 2.17 to 3.35% and 2.75 to 4.02% for rendered fat. Recovery of GTH, depending on spiked concentration, ranged from 80.47 to 91.98% for MBM and from 89.15 to 89.86% for rendered fat. The highest uncertainty values were 18.54% for MBM and 14.03% for rendered fat.

**Table 4.** Validation results of gas chromatography–flame ionisation detection method for glycerol triheptanoate measurement in animal by-product samples

Validation parameter	Validation result					
	Meat and bone meal				Fat	
Linearity	0.999					
R^2^	75–600					
Working range (mg/kg of fat)						
Selectivity	No interference					
Limit of detection (mg/kg of fat)		28.93			25.07	
Limit of quantification		60.43			37.26	
Matrix spiking level (mg/kg of fat)	75	250	600	75	250	600
Repeatability (CV, %)	5.20	8.79	2.64	3.35	2.17	2.95
Within-laboratory reproducibility (CV, %)	11.79	9.76	2.87	2.75	4.02	3.14
Recovery (%)	80.47	91.98	91.54	89.86	89.65	89.15
Uncertainty (U, %)	18.40	18.54	8.39	14.03	13.20	13.69

**Table 5.** Validation results of the gas chromatography–mass spectrometry method for glycerol triheptanoate measurement in animal by-product samples

Validation parameter	Validation result					
	Meat and bone meal				Fat	
Linearity						
R^2^	0.999					
Working range (mg/kg of fat)	75–625					
Selectivity	No interference					
Limit of detection (mg/kg of fat)		13.98			16.76	
Limit of quantification		21.68			23.66	
Matrix spiking level (mg/kg of fat)	75	250	625	75	250	625
Repeatability (CV, %)	2.03	3.89	3.68	8.97	2.22	3.53
Within-laboratory reproducibility (CV, %)	9.63	7.42	4.30	7.35	1.94	6.15
Recovery (%)	91.50	89.40	87.60	85.50	87.60	89.10
Uncertainty (U, %)	7.31	8.25	7.91	15.33	5.16	7.75

For GC-MS analysis, the LODs were 13.98 mg/kg of fat for MBM and 16.76 mg/kg of fat for rendered fat, and the LOQs were 21.68 mg/kg of fat and 23.66 mg/kg of fat, respectively. The coefficients of variation for repeatability ranged from 2.03 to 3.89% and for reproducibility from 4.30 to 9.63% for MBM, and these ranged from 2.22 to 8.97% and from 1.94 to 7.35% for rendered fat. Recovery of GTH, depending on spiked concentration, ranged from 87.60 to 91.50% for MBM and from 85.50 to 89.10% for rendered fat. The highest uncertainty was calculated as 8.25% for MBM and 15.33% for rendered fat.

Additionally, the methods were evaluated in international interlaboratory comparisons organised by the Centre Wallon de Recherches Agronomiques, Gembloux, Belgium in 2019 and Wageningen University and Research, Wageningen, the Netherlands in 2024. Correct results were obtained, and the *z*-scores values were within the range of –0.7–1.1 in 2019 and –0.16–0.09 in 2024.

### Sample analysis

**Fig. 3.**
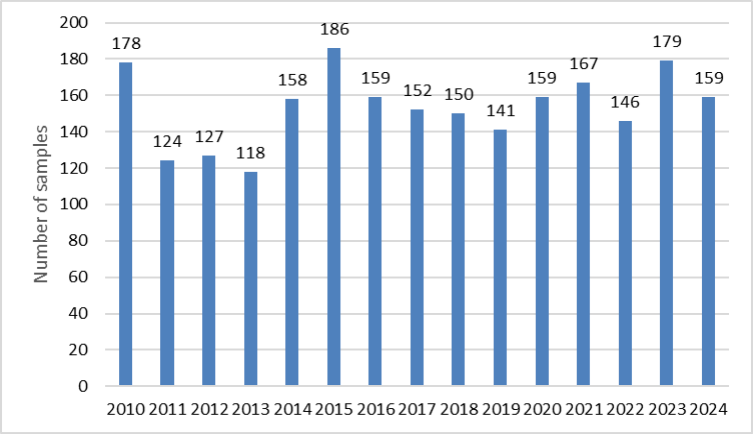
Number of animal by–products samples tested in 2010 – 2024

**Fig. 4.**
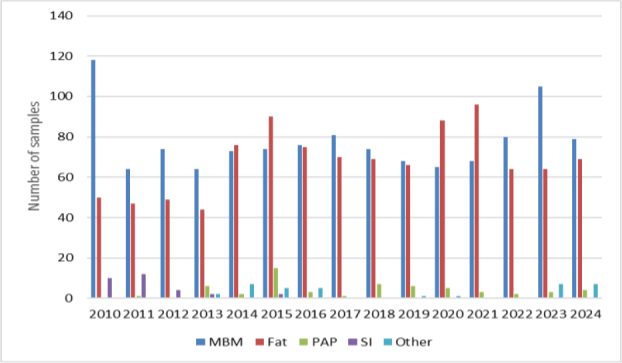
Number of animal by-product samples analysed in individual years by broad category of tested material. MBM – meat and bone meal; Fat – rendered fat; PAP – processed animal protein; SI – soil improver; Other – antioxidants, feed materials and mixtures, dog chews, feathers, bird balls, unknown material of animal origin and GTH markers

Optimised and validated methods were used for routine analyses. In 2010–2024, a total of 2,303 samples were tested, the most frequently tested materials being MBM and rendered fat. Among the analytes there were also GTH marker samples. Less common materials were also tested, including antioxidants, feathers and dog chews. The largest number of samples was tested in 2015 and was 186, and the smallest in 2013, when 118 were submitted. The annual distribution of tested samples is shown in Fig. 3. Between 2010 and 2013, MBM was the predominant material among the samples tested. From 2014 to 2021, this trend shifted, with rendered fat accounting for as many samples as MBM or more. No soil improver samples were received for testing after 2016. A list of samples and their broad categories analysed between 2010 and 2024 is provided in Fig. 4.

**Fig. 5.**
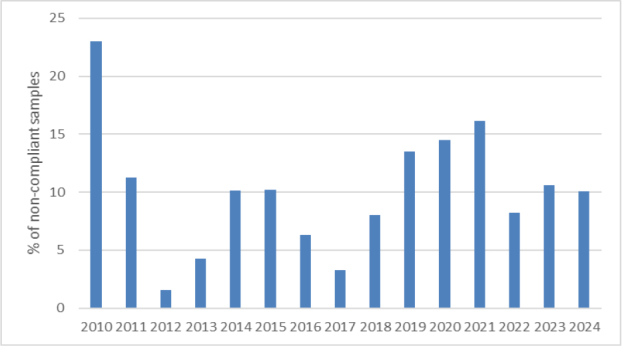
Percentage of non-compliant samples in individual years

Samples that did not meet the requirements under the applicable law, *i.e*. MBM and rendered fats of category 1 and 2 containing less than 250 mg of GTH per kg of fat and PAP and samples in the collective “others” category in which the presence of GTH was confirmed, accounted for approximately 10.5% (240 samples). The highest number of non-compliant samples was 41, recorded in 2010, which was as much as 23.2% of all samples tested that year. The lowest was 2, recorded in 2012, a mere 1.6%. The percentage of non-compliant samples in individual years is shown in Fig. 5. Considering material type, MBM had the lowest percentage of non-compliant samples at approximately 8%, while PAP samples in which GTH was detected accounted for almost 21% of all such samples tested. The concentrations of GTH determined in PAPs ranged from the LOQ up to 614 mg/kg of fat. Non-compliant samples in the groups of fat, soil improvers and other materials accounted for 12%, 10% and 14%, respectively.

## Discussion

Regulation (EC) No. 1432/2007 of 5 December 2007 introduced changes to the marking and transportation of animal by-products and became effective on 1 July 2008 (4). The marking procedures for animal by-product materials not intended for human consumption and posing a high risk to the food chain were clarified. Category 1 and 2 products were regulated most heavily, and had to be permanently marked, as they were and are prohibited from entering into the feed and food chain. Marking these products ensures the traceability of animal by-products destined for disposal and helps eliminate the risk of fraud or adulteration. Consequently, it became necessary to develop or optimise methods for determining the GTH marker in materials derived from the processing of category-1 and -2 ABPs.

The literature on the subject is very limited, with publications available from only two research centres the EU Commission Directorate General Joint Research Centre Institute for Reference Materials and Measurements in Geel, Belgium and the Instituto Zooprofilattico Sperimentale del Pielmonte in Torino, Italy (1, 2, 13, 14, 16, 17). This literature described good separation of signals from GTH and the internal standard being achieved using non-polar or medium-polarity capillary columns. In our study, the best results were obtained using non-polar capillary columns. The use of helium as the carrier gas and the specification of the flow rates for both methods correlated with the literature data. The rise from the initial to the final temperature of the thermostat should take no longer than 15 min and temperature increases should be appropriately timed to obtain chromatograms with good signal separation from the analysed substances and to eliminate potential interference, according to the literature on GTH detection gas chromatography temperature programmes. Neither our GC-FID nor GC-MS programmes exceeded this time and our signal separations were distinct. The reported data which were consulted indicated that injector temperatures ranged from 250°C to 300°C, and samples could be injected either in splitless mode or with a split ratio of up to 50:1. Our injection volume for both detection modes was 2 μL, the temperatures fell in the range and the split ratios were well below the noted maximum, thus being consistent with the literature (2, 16). The best FID results were obtained with the detector operating temperature set to 360°C and a hydrogen:air ratio of 1:10. In the case of the MS method, the ionisation type, spectral acquisition method, m/z ratio of the ions of the analysed substances and the ion source temperature were optimised. No significant differences were introduced from the published data for MS (13, 15).

The methods’ validation results indicated that they met the criteria for linearity, recovery, precision, and detection capability. The linearity data obtained during our own studies demonstrated their accuracy, as confirmed by a correlation coefficient greater than 0.99. The chromatograms show no interfering peaks originating from the matrix with retention times close to or overlapping with the analysed substances, demonstrating the selectivity of the methods (Figs 1a and 2a). Available literature data indicate recoveries ranging from 95.6% to 101%, depending on the matrix fortification level (16). Our recoveries ranged from 89.15% to 89.86% for rendered fat samples and from 80.47% to 91.98% for meat and bone meal samples for the GC-FID method and from 85.50% to 89.10% and from 87.60% to 91.50% for the GC-MS method, respectively. The coefficients of variation for the analysed substance were comparable regardless of the detector used. In the case of fat as a matrix, the coefficients ranged from 2.17% to 3.35% for repeatability and from 2.75% to 4.02% for within-laboratory reproducibility for GC-FID, and from 2.22% to 8.97% and from 1.94% to 7.35% for GC-MS, respectively. For MBM, these values ranged from 2.64% to 8.79% for repeatability and from 2.87% to 11.79% for within-laboratory reproducibility in case of GC-FID method, and from 2.03% to 3.89% and from 4.30% to 9.63% in case of GC-MS method, respectively. The limits of detection and quantification for the methodological approaches used in these tests and described in the available literature were published as 4.8 and 16 mg/kg of fat, or 20 and 41.6 mg/kg of fat basis, respectively (2, 16). However, these limits were only applicable to the GC-MS method. The FID validation phase produced LOD and LOQ values for rendered fat of 25.07 and 37.26 mg/kg of fat, respectively, and this phase for MS yielded 16.76 and 23.66 mg/kg of fat. For MBM, these values were 28.93 and 60.43 mg/kg of fat, respectively for FID, and 13.98 and 21.68 mg/kg of fat for MS. As expected given the higher sensitivity of MS, lower LOD and LOQ values were obtained with MS detection than with FID. The maximum expanded uncertainty values for the discussed methods were 15.33% for rendered fat and 18.54% for MBM.

The results of the real sample analyses highlight the effectiveness of the optimised and validated methods for routine monitoring of animal by-products. Over the 15-year period, the majority of tested materials were MBM and rendered fat, reflecting the highest-volume streams in the rendering industry. The shift observed after 2014, with rendered fat becoming as frequent as or more frequent than MBM, may indicate changes in production patterns, regulatory focus or sample submission practices.

The overall proportion of non-compliant samples (10.5%) suggests that most materials on the market complied with legal requirements. However, the relatively high proportion of non-compliance in PAP (21%) compared with that of MBM (8%) points to greater risks in PAP handling or processing. The out-of-range sample proportion observed in rendered fat (12%), soil improvers (10%) and other materials (14%) underlines the importance of continuous monitoring across all categories and varied derived products. The relatively high percentage of non-compliant samples may be due to the difficulty in estimating the appropriate amount of marker to be added to the processed material to achieve the desired concentration in processed products. While the fat content of the processed material is difficult to determine, the GTH content in the derived product is related to it and should be expressed on a fat weight basis.

It proved challenging to compare our routine sample analysis results with data from other EU countries. The work of the expert group at the European Reference Laboratory for Processed Animal Protein (EURL-AP) drew in reports from only a limited number of laboratories on the annual number of samples analysed, which ranged from as few as 10 to more than 500. The only published dataset covers the years 2017–2021 and includes 167 samples of category-1 and -2 MBM, category-3 fat, and PAP (14). These results showed that approximately 10% of samples failed to meet the specified requirements, a finding consistent with our study.

The availability of reliable methods for determining GTH will be crucial in ensuring food and feed safety, particularly in light of the recent relaxation of the ban on the use of MBM and PAP. The ability to detect category-1 and -2 products is essential to prevent adulteration and the introduction of high-risk materials into the feed chain. Future work should focus on harmonising analytical practices across laboratories; expanding surveillance to cover a broader range of animal by-products and feed; and investigating complementary markers and rapid detection methods to further strengthen the monitoring framework.

## Conclusion

The conducted studies demonstrated optimised and validated analytical methods based on gas chromatography with flame ionisation detection or mass spectrometry detection for GTH. Determination of the GTH marker can be claimed to be an effective tool for monitoring and enforcement of ABP regulations. These methods ensure traceability, facilitate the detection of fraud and adulteration and help safeguard the integrity of the feed and food chain. Importantly, the findings show that when applied consistently, validated analytical approaches provide a solid foundation for regulatory decision-making and public health protection. Routine testing carried out between 2010 and 2024 using the optimised methods revealed that the vast majority of materials (approximately 90%) were either correctly marked with GTH or free of the compound as required.
